# Nickel and cobalt resistance properties of *Sinorhizobium meliloti* isolated from *Medicago lupulina* growing in gold mine tailing

**DOI:** 10.7717/peerj.5202

**Published:** 2018-07-10

**Authors:** Zhefei Li, Xiuyong Song, Juanjuan Wang, Xiaoli Bai, Engting Gao, Gehong Wei

**Affiliations:** 1 Shaanxi Key Laboratory of Agricultural and Environmental Microbiology, College of Life Science, Northwest A&F University, Yangling, Shannxi, China; 2 Northwest A and F University, State Key Laboratory of Crop Stress Biology in Arid Areas, Yangling, Shaanxi, China

**Keywords:** *Sinorhizobium meliloti*, Nickel, Cobalt, Resistance, Cation transporter

## Abstract

*Sinorhizobium meliloti* CCNWSX0020, isolated from root nodules of *Medicago lupulina* growing in gold mine tailings in the northwest of China, displayed multiple heavy metal resistance and growth promotion of *M. lupulina*. In our previous work, the expression level of *dmeR* and *dmeF* genes were induced by Cu^2+^ through comparative transcriptome approach. Based on protein analysis, the *dme*F encoded for a protein which showed a 37% similarity to the cation transporter DmeF of *Cupriavidus metallidurans*, whereas *dme*R encoded transcriptional regulator which was highly homologous with DmeR belonging to RcnR/CsoR family metal-responsive transcriptional regulator. In addition to copper, quantitative real-time PCR analysis showed that *dme*R and *dme*F were also induced by nickel and cobalt. To investigate the functions of *dme*R and *dme*F in *S. meliloti* CCNWSX0020, the *dme*R and *dme*F deletion mutants were constructed. The *dme*F mutant was more sensitive to Co^2 +^ and Ni^2 +^ than the wild type strain. Pot experiments were carried out to determine whether the growth of *M. lupulina* was affected when the *dmeF* gene was knocked out in the presence of nickel or cobalt. Results indicated that the nodule number of the host plant inoculated with the *dme*F deletion mutant was significantly less than the *S. meliloti* CCNWSX0020 wild-type in the presence of Co^2 +^ or Ni^2 +^. However, when standardized by nodule fresh weight, the nitrogenase activities of nodules infected by the *dme*F deletion mutant was similar to nitrogenase activity of the wild type nodule.

## Introduction

Heavy metals are one of most common components in the environment. Some metals do not have a clear physiological function and are toxic to microorganisms even at low concentrations, such as lead and cadmium. Some heavy metals, such as cobalt, copper and nickel, are fundamental elements for living organisms and are involved in many physiological processes. In particular, cobalt is required for vitamin B12-dependent enzymes and proteins (Banerjee & Ragsdale, 2003), whereas nickel acts as a metal cofactor for some enzymes (Mulrooney & Hausinger, 2003; Guldan, Sterner & Babinger, 2008). However, in addition to being involved in some metabolic processes, an excess of cobalt and nickel can also damage cells by producing reactive oxygen species (Ahmad et al., 2015; Haferburg & Kothe, 2007; Liu et al., 2015). Therefore, releasing cobalt and nickel due to industrial and mining operations poses a significant threat to living organisms.

To prevent intracellular cobalt and nickel overload-mediated toxicity, many microorganisms have developed several mechanisms to protect themselves from an excess of metals (Rutherford, Cavet & Robinson, 1999; Pini et al., 2014; Matuszewska et al., 2008; Nies, 1999). Under normal conditions, these microelements are transported into cells. However, specific ion efflux systems have been used to eliminate excess metal ions from the cytoplasm. Therefore, efflux systems, uptake systems, the synthesis of ligand compounds and metallochaperones for regulating cobalt and nickel homeostasis play a crucial role in most cobalt/nickel-resistant organisms. Among these cobalt/nickel-resistant systems, resistance nodulation cell division efflux pumps (RND) (Stahler et al., 2006), cation diffusion facilitators (CDF) (Dokpikul et al., 2016) and *P*_1b_-type ATPases have been highlighted (Rutherford, Cavet & Robinson, 1999).

The first characterized CDF protein was CzcD, which was shown to participate in heavy metal tolerance in *Cupriavidus metallidurans* (Paulsen & Saier Jr, 1997). Later reports said that CDF family proteins were found to be ubiquitous in all living organisms (Nies, 2003). Proteins belonging to the cation diffusion facilitator (CDF) family have been implicated in metal tolerance. Most CDFs were located on internal membranes and catalysed the efflux of transition metal cations, including Zn^2+^, Co^2+^, Fe^2+^, Cd^2+^, Ni^2+^, or Mn^2+^, from the cytoplasm to the outside of the cell (Kolajrobin et al., 2015). Based on phylogenetic analysis, the CDF family was divided into Mn^2+^-transporting CDF, Fe^2+^/Zn^2+^-transporting CDF, Zn^2+^ and other metal transporting CDF according to the metal ion specificity (Montanini et al., 2007). The majority of CDF proteins from diverse sources have the following features in common: (1) they possess six putative transmembrane domains (TMDs) and share a signature sequence between TMD1 and TMD2; and (2) they share a C-terminal cation efflux domain. Many CDF transporters also contain a histidine-rich domain. Such domains are predicted to allow more efficient metal binding. In addition, it has been suggested that bacterial CDFs may participate in other biological functions. For instance, CepA confers chlorhexidine resistance to *Keumonia* (Fang et al., 2002). MamB and MamM of *Magnetospirillum gryphiswaldense* have been linked to magnetosome formation (Uebe et al., 2011). *Sinorhizobium meliloti* is a widely investigated model rhizobium species for symbiosis with leguminous plants. However, high concentrations of heavy metals have adverse effects on the rhizobia population (Sharaff & Archana, 2015; Wani, Khan & Zaidi, 2008). Our laboratory team recently isolated and sequenced the genome of heavy metal-resistant *S. meliloti* CCNWSX0020 that significantly improved the growth of *Medicago lupulina* in copper-contaminated soil. The genomic sequence data gave us information about the genes encoding putative proteins involved in heavy metal resistance in *Sinorhizobium*. So far, there is scarce functional evidence about genetically determined mechanisms of cobalt and nickel resistance in *S. meliloti* CCNWSX0020. In this study, molecular determinants responsible for cobalt and nickel resistance in *S. meliloti* were investigated. We also investigated whether cobalt- and nickel-sensitive mutants generated by homologous recombination affected the symbiotic nodulation capability with the host plant under cobalt or nickel stress conditions.

**Table 1:** Bacteria, plasmids and primers used in the work

Bacteria, plasmids or primer	Features	Source
**Strains**		
*S. meliloti* CNWSX0020	Wild type, Amp^r^	Fan et al. (2011)
*E. coli* DH5a	lacZ4M15, recA1, gyrA96, hsdR17	Hanahan (1983)
SM0020 ΔdmeF	*dme*F deleted in *S. meliloti* CNWSX0020	This work
SM0020 ΔdmeR	*dme*R deleted in *S. meliloti* CNWSX0020	This work
**Plasmids**		
pk18 mob sacB	Suicide vector, Mob^+^, Km^r^	Schafer et al. (1994)
pBBR1MCS-5	Broad-host-range cloning vector, Gmr	Kovach et al. (1995)
pRK2013	Helper pasmid, Km^r^	University of York, Prof. Tanya Soule
pK18-ΔdmeR	Containing *dme*R deletion fragment	This study
pK18-ΔdmeF	Containing *dme*F deletion fragment	This study
pBBR-dmeF	pBBR1MCS-5 contain entire *dme*F	This study
pBBR-dmeR	pBBR1MCS-5 contain entire *dme*R	This study
**Primers**		
dmeFF1	CGGGATCCTTGGCACCAGAAAGAAGACGA	
dmeFR1	GCTATGGTGGTGCTCGTGATGCCATCATTCC	
	CGCAGTCAGT	
dmeFF2	ACTGACTGCGGGAATGATGGCATCACGAG	
	CACCACCATAGC	
dmeFR2	GCTCTAGATCCTCTTCCGCATTCACGAC	
dmeRF1	CGGGATCCAAGCCGCGACTGGGAAGA	
dmeRR1	TCTCCCTGGGTTTCGTGGGGAGGCGACG	
	AGGTTGAGA	
dmeRF2	TCTCAACCTCGTCGCCTCCCCACGAAAC	
	CCAGGGAGA	
dmeRR2	GCTCTAGAGCAGAGCGATCAAGGCAAGTA	
dmeH1	ATCCCGGGGTTTGGCACCAGAAAGAAGACGA	
dmeH2	GCTCTAGAGCAGAATGCAGCCGCTAAGAT	

**Notes.**

Underlined indicates the restriction site.

## Materials & Methods

### Bacterial strains, media and growth conditions

All bacterial strains and plasmids used in this study are listed in Table 1. *Escherichia coli* DH5 *α* was grown in Luria-Bertani (LB) medium at 37 °C. *S. meliloti* CCNWSX0020 and the mutants were grown at 28 °C in tryptone-yeast extract medium (TY medium: 5 g tryptone, 3 g yeast extract, 0.7 g CaCl_2_ ⋅2 H_2_O and 15 g agar per litre). Liquid culture of the cells was carried out in shaken tubes or Erlenmeyer flasks at 180 rpm. When necessary, media were supplemented with 100 µg/mL ampicillin (Amp), 50 µg/mL kanamycin (Km) or 50 µg/mL gentamicin (Gm).

### qRT-PCR assays

*S. meliloti* CCNWSX0020 was cultured to logarithmic phase in TY liquid medium. Different heavy metals were added to the logarithmic phase medium, and the final concentrations of CoCl_2_, NiCl_2_, CuSO_4_, ZnCl_2_, Pb(NO_3_) _2_ andCdCl_2_ were adjusted to 0.3, 0.5, 0.5, 0.5, 0.5 and 0.2 mM. Then, culture medium was incubated for 15 min before the total RNA was extracted. Residual DNA in the total RNA was removed by DNase I. A TakaRa reverse transcription kit and SYBR Premix ExTaqTM II (Tli RNaseH Plus) kit were used for reverse transcription and qRT-PCR. All experimental operations were carried out according to the manufacturer’s instructions. To standardize the results, 16S rRNA was used as an internal standard and the relative levels of transcription were calculated using the 2^−ΔΔ*Ct*^method (Livak & Schmittgen, 2001).

### Bioinformation analyses

The draft genome of *S. meliloti* CCNWSX0020 (AGVV00000000) was previously sequenced and deposited in GenBank. The known DmeF protein sequences of most bacterial genomes used in this study were obtained from NCBI (https://www.ncbi.nlm.nih.gov). The whole set of bacterial DmeF sequences was aligned using ClustalW2 and the phylogenetic tree visualized with MEGA 6.0 (http://www.megasoftware.net). The DmeF membrane topology of strain CCNWSX0020 was generated and visualized by HMMTOP (version 2.0; http://www.enzim.hu/hmmtop/).

### Generation of deletion mutants in *dme*R and *dme*F

**Figure 1:**
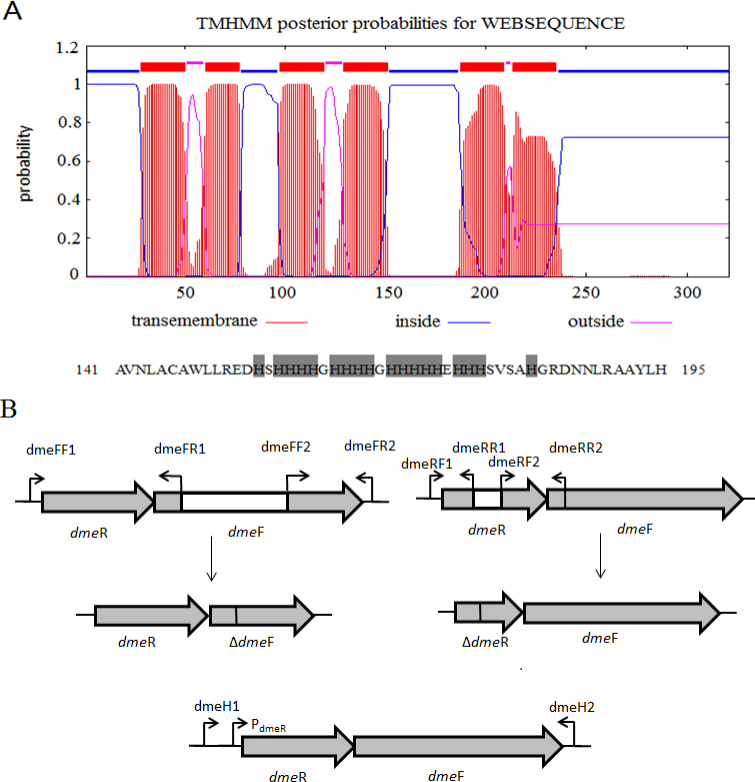
Gene organization of *S. meliloti* CCNWSX0020 *dme*RF and transmembrane structure of. (A) Predicted transmembrane structure of DmeF, a histidine-rich stretch locates between TMD4 and TMD5 of DmeF. (B) Gene organization of *S. meliloti* CCNWSX0020 *dme*RF. The *dme*F** (SM0020_17742) is located downstream of the *dme*R (SM0020_17737). White region represents deletion sequence; *P*_*dme*R_ represents *dme*R promoter.

The total genomic DNA of *S. meliloti* CCNWSX0020 was extracted according to the protocol of Wilson & Carson (2001). A 940-bp upstream and a 590-bp downstream fragment of *dme*F were amplified using the primer pairs dmeFF1/dmeFR1 and dmeFF2/dmeFR2, respectively. The upstream and downstream PCR products were ligated by crossover PCR with primer pairs dmeFF1/dmeFR2 (Fig. 1B). The resulting 1.53-kb fragment was digested with *Bam* HI/*Xba*I and cloned into the *Bam* HI/*Xba*I site of the suicide vector pK18mobsacB to produce pK18-ΔdmeF. For the construction of pK18-ΔdmeR, 455-bp upstream and 405-bp downstream fragments of *dme*R were amplified using the primer pairs dmeRF1/dmeRR1 and dmeRF2/dmeRR2, respectively. The upstream and downstream PCR products were ligated by crossover PCR with primers dmeRF1/dmeRR2. The resulting 860-bp fragment was digested with BamHI/XbaI and cloned into the BamHI/XbaI site of the suicide vector pK18mobsacB. All primers used in this study are listed in Table 1. The constructed suicide plasmid pK18-ΔdmeF or pK18-ΔdmeR was transferred into *S. meliloti* CCNWSX0020 by triparental mating, which included *S. meliloti* CCNWSX0020 (Amp ^r^) as the recipient, *E. coli* JM109 cells containing pK18- Δ*dme*F (Km^r^) as the donor, and *E. coli* DH5*α* cells containing pRK2013 as helper cells. A single clone of transferred *S. meliloti* CCNWSX0020, which was resistant to both kanamycin and ampicillin, was grown in TY solid medium containing ampicillin and 10% (w/v) sucrose. Double crossover recombinants were confirmed by PCR using dmeFF1 and dmeFR2 as primers, and then the correct PCR products were sequenced. The resulting mutants were designated as SM0020Δ*dme*F and SM0020Δ*dme*R (Table 1).

### *dme*F/*dme*R deletion mutant complementation experiment

To complement the *dme*F and *dme*R mutants, the entire *dme*RF including the *dme*R promoter was amplified from *S. meliloti* CCNWSX0020 with primers dmeH1/dmeH2 (Fig. 1B). The PCR products were digested with *Sma*I/*Xba*I and inserted into a broad-range plasmid pBBR1MCS-5 to generate pBBR-dmeF. The complement plasmids were transformed into SM0020 Δ*dme*F, and single clones harbouring pBBR-dmeF were selected on TY solid medium supplemented with 50 µg/mL Gm. The presence of the entire *dme*F gene in the mutant strain was confirmed by PCR.

### Determination of the metal sensitivity of the defective mutant

Heavy metal sensitive assays of CCNWSX0020 strain, SM0020 Δ*dme*F and the complementary strain were carried out on TY solid medium. The wild-type strain and *dme*F mutant were grown to mid-exponential phase in TY liquid medium at 28 °C with shaking at 150 rpm. Cells were grown to the exponential phase in TY liquid medium and diluted to an OD600 of 0.1. Five 10-fold dilutions were spotted on the TY solid medium and incubated at 28 °C for 24 h. Each experiment was repeated three times.

### Determination of Maximum Tolerable Concentrations (MTCs)

*S. meliloti* CCNWSX0020, *dme*F mutant and *dme*F mutant carrying pBBR-dmeF plasmid were grown to mid-exponential phase in TY liquid medium at 28 °C with shaking at 200 rpm, and cell suspensions were prepared at the same OD600 of 1.0 (optical density at 600 nm). Then, 1% of the cell suspensions was added to fresh TY medium supplemented with a different concentration of CuCl_2_, ZnCl_2_, CoCl_2_ and NiCl_2_. The cells were incubated with shaking at 200 rpm for 48 h, and the growth was monitored at OD600. The data are shown as the means of biological triplicates±SD.

### Plant tests

*M. lupulina* seeds were surface sterilized and germinated in petri dishes with water agar (5 g agar per litre) at 28 °C for 48 h, and then seedlings were sown in pots filled with sterilized perlite-vermiculite (3:2) supplemented with different concentrations of CoCl_2_ or NiCl_2_ and grown in a greenhouse at 25 °C. When the first main leaf grew out, suspensions of either *S. meliloti* CCNWSX0020 or *dme*F mutant were added to each plant root with a final concentration of 10^8^ CFU per root. Plants were harvested 21 days after inoculation, the number of nodules on the plant roots was counted, and the lengths of the shoots and roots were measured. Nitrogenase activity in nodules was measured by the acetylene reduction assay as described by Hardy et al. (1968). Fresh nodules from *M. lupulina* inoculated with *S. meliloti* CCNWSX0020 and the *dme*F mutant in the presence of 100 mg/kg cobalt or nickel were fixed in FAA solution (90 mL 70% ethanol, 5 mL acetic acid, and 5 mL 40% methanol) for 16 h. Dehydrating and clearing processing were carried out through a graded ethanol series and chloroform series, respectively, followed by embedding and sectioning of the paraffin blocks. Paraffin-embedded nodule sections of 5–10-µm thickness were stained by 0.05% (w/v) toluidine blue solution for observation with a BX53 biological microscope (Olympus, Tokyo, Japan).

### Statistical analysis

SPSS 18.0 (SPSS Inc., Chicago, IL, USA) was used for statistical analysis of the data. Data were compared by analysis of variance and multiple comparison tests.

## Results

### Identification of nickel- and cobalt-resistant genes

*S. meliloti* CCNWSX0020, isolated from the root nodules of *M. lupulina* growing in gold mine tailings in northwest China, could be resistant to many types of heavy metals, such as Cu^2+^, Zn^2+^, Pb^2+^ and Cd^2+^ (Fan et al., 2011). In our previous work, we found that the expression of two putative genes SM0020-17742 and SM0020-17737 was induced by Cu^2+^ through transcriptome sequencing. The 966-bp-long open reading frame of SM0020-17742 encodes a 321-amino-acid protein, and the deduced protein shows high identity with several previously characterized cobalt- and nickel-resistant proteins: DmeF of *C. metallidurans* (ABF07084, 37%) and DmeF of *A. fabrum* (AAK86697, 52%), so we designated the SM0020-17742 gene *dme*F. In *C. metallidurans* and *A. tumefaciens*, the DmeF protein has an important role in cobalt and nickel resistance (Dokpikul et al., 2016; Munkelt, Grass & Nies, 2004). However, the phylogenetic tree based on the DmeF protein sequence showed that the DmeF proteins of *S. meliloti* CCNWSX0020, *S. arboris* and *S. medicae* are more closely related to each other than that of *C. metallidurans* and *A. tumefaciens* (Fig. S1). DmeF of *S. meliloti SM0020* contained six predicted transmembrane segments (http://www.cbs.dtu.dk/services/TMHMM), with the histidine-rich stretch located between TMD4 and TMD5 (Fig. 1A). Another gene, SM0020_17737, is located directly upstream of *dme*F (SM0020_17742) and predicted to be in the same operon with *dme*F (Fig. 1B). SM0020_17737 encodes a 90-amino-acid protein that is highly homologous with DmeR belonging to the RcnR/CsoR family of metal-responsive transcriptional regulators. *E. coli* RncR binds to the *rncA* promoter DNA fragment in the absence of Ni^2+^ or Co^2+^, and the affinity of RncR for this promoter is reduced in the presence of excess nickel or cobalt. Alignment of sequences revealed that the upstream region of SM0020_17737 contains an inverted repeat (*ATAGGGTACCCCCCTATGCTATG*) between -35 and -10 similar to the *dme*RF promoter of *A. tumefaciens* (Dokpikul et al., 2016). These observations suggest that the expression of both SM0020_17737 and SM0020_17742 (*dme*F) might be regulated by the SM0020_17737 gene product, so the SM0020_17737 gene was designated as *dme*R.

**Figure 2:**
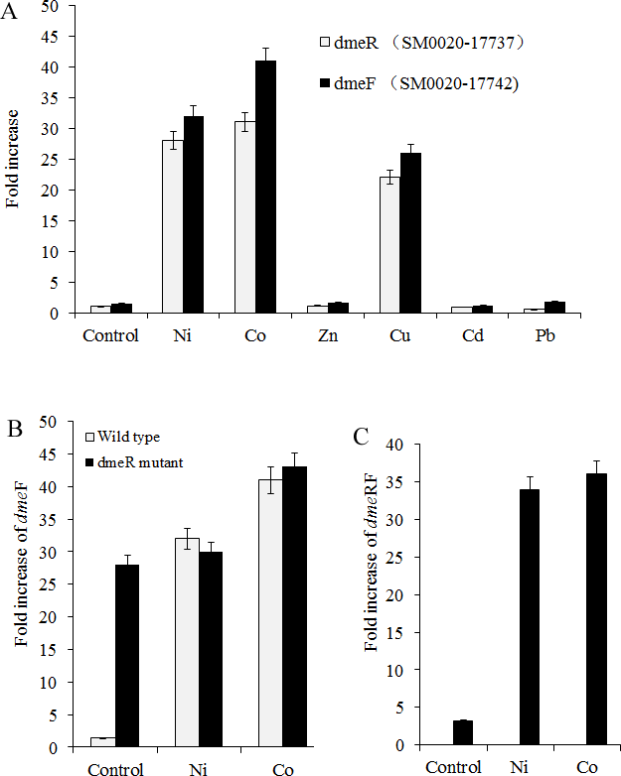
dmeFR PCR analysis. Induction of *dme*F and *dme*R of wild type or *dme*R mutant** by various metals examined through quantitative real-time PCR analysis. Wild type and *dme*R mutants of *S. meliloti* CCNWSX0020 strains at OD_600_ of 1.0 were incubated with 0.5 mM CuSO_4_, ZnCl_2_, Pb(NO_3_)_2_, NiCl_2_, 0.2 mM CdCl_2_ and 0.3 mM CoCl_2_ for 30 min. The fold changes in *dme*F and *dem*R** expression are expressed relative to the untreated control. Samples were then processed for qPCR analysis and normalized against the ribosomal 16 S rRNA. Error bars represent standard deviations of three biological repeats.

### Nickel and cobalt induced dmeRF gene transcription in *S. meliloti* CCNWSX0020

Since heavy metal efflux systems of other bacteria are activated in the presence of the corresponding metal cation, we decided to investigate which metal could affect the expression of the *dme*R and *dme*F genes in *S. meliloti* CCNWSX0020 besides Cu^2+^. Expression of the *dme* R and *dme*F** genes was analysed first in free-living cells from *S. meliloti* CCNWSX0020 under different metal stresses. The expression of *dme*F was strongly up-regulated by Ni^2+^, Co^2+^ and Cu^2+^ exposure (∼30-fold for 0.5 mM Ni^2+^, ∼40-fold for 0.3 mM Co^2+^ and ∼25-fold for 0.5 mM Cu^2+^), while the expression of *dme*R was induced by Ni^2+^ (30-fold), Co^2+^ (30-fold) and Cu^2+^ (20-fold) (Fig. 2A). No significant induction of *dme*R and *dme*F was observed when Zn^2+^, Pb^2+^, or Cd^2+^ was added at concentrations up to 0.5 mM (Cd^2+^, 0.2 mM). The *dme*R gene was located directly upstream of *dme*F, and deletion of *dme*R led to enhancement of the *dme*F gene expression with or without nickel/cobalt stresses; meanwhile, the expression of *dme* RF was increased by cobalt and nickel treatment (Figs. 2B and 2C). These results suggested that DmeR is a cobalt/nickel sensor and regulates the expression of *dme*F and its own.

### Functional analysis of *dme*F in the CCNWSX0020 strain

**Figure 3:**
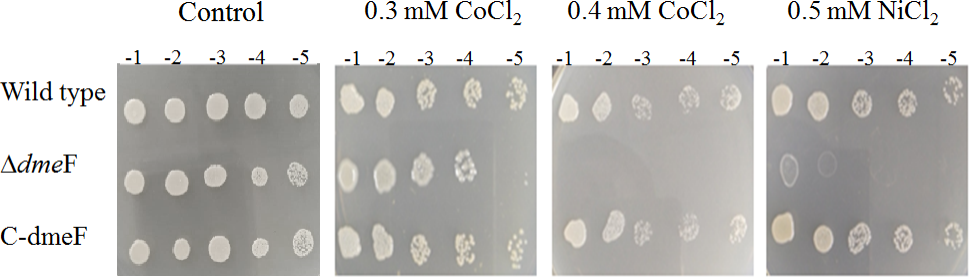
Sensitivity of wild type and *dme*F mutant to cobalt/nickel. Sensitivity of wild type, *dme*F mutant (Δ *dme*F) and complemented strains (C- *dme*F) of *S. meliloti* CCNWSX0020 to cobalt and nickel. Log-phase cells grown in TY were adjusted, serially 10-fold diluted and spotted onto TY plates in the presence of the indicated concentrations of CoCl_2_ (0.3 mM or 0.4 mM) and NiCl_2_ (0.5 mM).

To determine the function of DmeF in *S. meliloti CCNWSX0020*, the *dme*F mutants were constructed by homologous recombination, resulting in strain SM0020Δ*dme*F. Since the DmeF protein, belonging to a cation diffusion facilitator, was responsible for resistance to nickel and cobalt in *A. tumefaciens* C58 (Dokpikul et al., 2016), the sensitivities of *S. meliloti CCNWSX0020* and *dme*F mutant were characterized using metal-tolerance growth assays in TY solid medium. The *dme*F mutant was more sensitive to 0.3 mM CoCl_2_ (10-fold) and 0.5 mM NiCl_2_ (10^3^-fold) than the wild type, and the growth of the *dme*F mutant was completely inhibited by 0.4 mM CoCl_2_ (Fig. 3). However, the resistance of the *dme*F mutant to other metals, including CuSO_4_, CdCl_2_, Pb(NO_3_)_2_, and ZnCl_2_, was similar to the wild type. Surprisingly, the gene expression of *dme*F was strongly induced by 0.5 mM CuSO_4_ (Fig. 2A), but there was no difference in the growth of the *dme*F mutant and wild type under copper stress. It is probable that Cu^2+^ could bind to DmeR and induce the expression of the *dme*RF operon, but DmeF was only the specific transporter of Co^2+^ and Ni^2+^. To verify the presence of the *dme*F genes that were responsible for cobalt and nickel resistance, the *dme*F PCR fragments containing the 550-bp upstream sequence of *dme*R were inserted into the pBBR1MCS-5 vector, transformed into the corresponding mutant and then tested for these metal tolerances. Figure 3 shows that complemented strains could restore the cobalt and nickel resistance of the mutants. These results demonstrated that DmeF plays an important role in resistance to cobalt and nickel in *S. meliloti* CCNWSX0020.

### Maximum tolerable concentration of the *dme*F mutant

**Figure 4:**
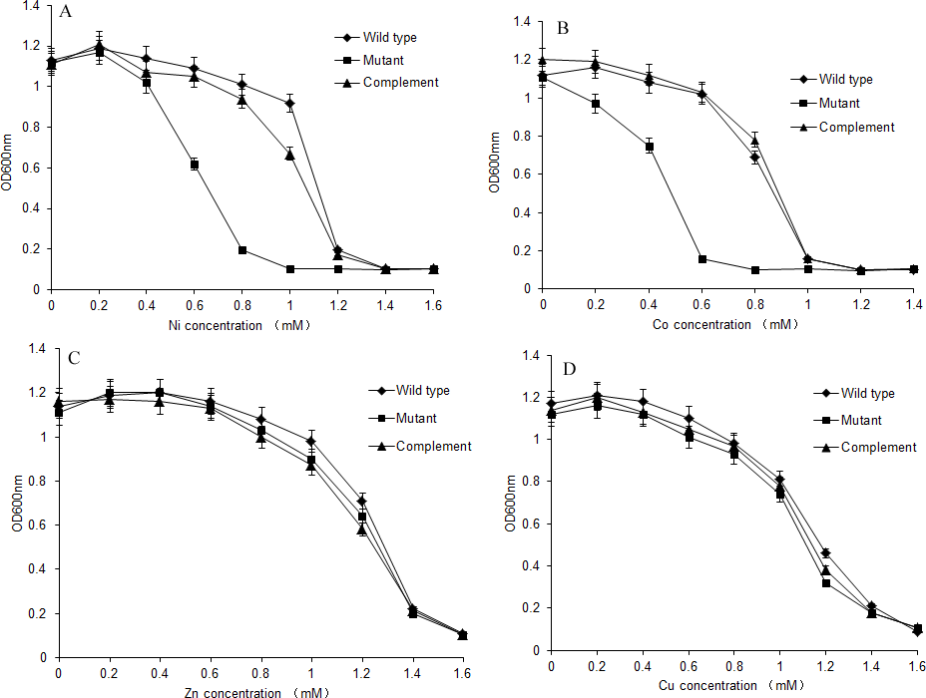
Growth of *S. meliloti* CCNWSX0020 and *dme*F mutant under different metal stress. Growth of *S. meliloti* CCNWSX0020 and the *dme*F mutant which had been incubated with the different heavy metals in TY medium for 48 h. (A) NiCl_2_; (B) CoCl_2_; (C) CuSO_4_; (D) ZnCl_2_. *S. meliloti* CCNWSX0020 wild type (⧫), *dme*F mutant(■), *dme*F mutant carrying pBBR-dmeF plasmid (▴).

*S. meliloti* CCNWSX0020 and the *dme*F mutant were cultured in TY medium supplemented with increasing concentrations of CoCl_2_, NiCl_2_, ZnCl_2_ and CuSO_4_, and the growth of the wild type and *dme*F mutant was analysed after 48 h. As shown in Fig. 4, the *dme*F mutant exhibited sensitivity to different concentrations of Co^2+^ and Ni^2+^ but not to other metals. The growth of the *dme*F mutant was significantly inhibited if the concentration of nickel or cobalt was higher than 0.8 mM or 0.6 mM, respectively. The maximum tolerances of the *S. meliloti CCNWSX0020* wild type to Co^2+^ and Ni^2+^ were 1.0 mM and 1.2 mM in TY liquid medium. In contrast, ZnCl_2_ and CuCl_2_ had no obvious effect on the growth of the wild type or mutant.

### Deletions of *dme*F decreased nodule number

**Figure 5:**
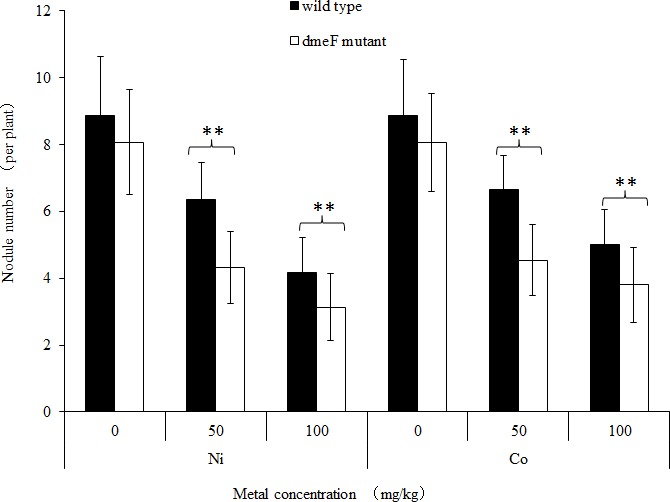
Influence of deletions in *dme*F on symbiosis. Influence of deletions in *dme*F on symbiotic nodulation. *M. lupulina* seedlings were sown in pots supplied with NiCl_2_ (A) or CoCl_2_ (B). The nodule number, length of roots and shoots were determined at 21 DAI.

To determine the effects of *dme*F on the symbiotic capacity of *S. meliloti* CCNWSX0020, *M. lupulina* seedlings were inoculated with the wild-type strain CCNWSX0020 or the *dme*F mutant. The plant length, nodule numbers and nitrogenase activities were determined to evaluate the symbiotic efficiency. Both *S. meliloti* CCNWSX0020 and the *dme*F mutant can form well-defined rod-shaped pink nodules with *M. lupulina*. No significant difference was observed in the nodule number between the wild-type strain and the *dme*F mutant without Ni^2+^ or Co^2+^ stress. However, the number of nodules produced by the *dme*F mutant decreased significantly (*P* < 0.01) compared to the nodule numbers generated by the wild-type strain under Ni^2+^ or Co^2+^ stress (Fig. 5).

**Figure 6:**
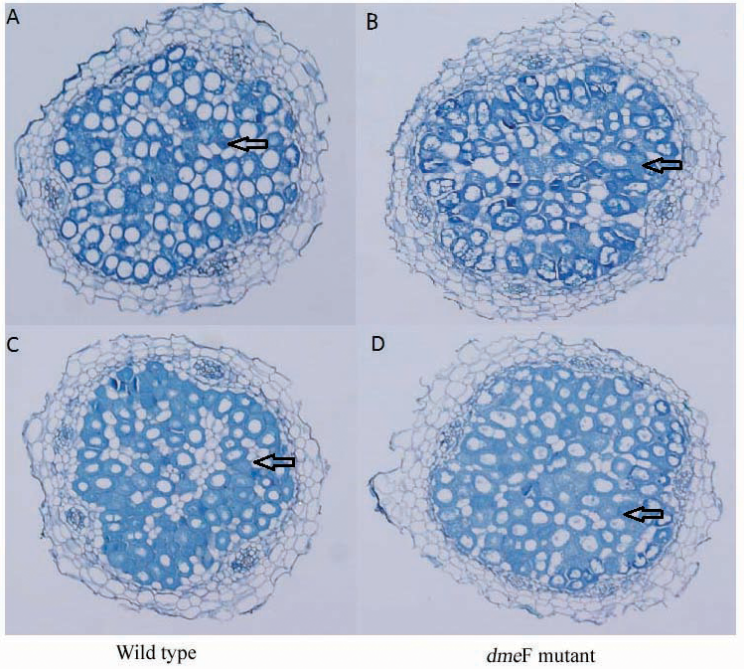
Light micrographs of nodule sections produced by *S. meliloti* wild-type strain and *dme*F mutant. Light micrographs of nodule sections produced by *S. meliloti* CCNWSX0020 wild-type strain and the *dme*F mutant with 100 mg/kg nickel (A and B) or cobalt (C and D).

For single metal treatment, the nodule number of the plant inoculated with the *dme*F mutant decreased by about 32.2% or 24.9% compared to the wild-type strain under 50 mg/kg or 100 mg/kg nickel stress, respectively. The same trend was observed when perlite-vermiculite were supplemented with CoCl_2_. The nodule number of *M. lupulina* inoculated with the *dme*F mutant was about 31.9% or 24% less than those inoculated with *S. meliloti* CCNWSX0020 in the presence of 50 mg/kg or 100 mg/kg CoCl_2_, respectively. No significant decreases (*P* < 0.05) in the root and shoot length of *M. lupulina* inoculated with the *dme*F mutant were observed compared with those inoculated with *S. meliloti* CCNWSX0020 in the presence of Ni^2+^ or Co^2+^ (Fig. S2). Since the nodulation of plants inoculated with the *dme*F mutant showed a significant reduction by treatment with CoCl_2_ and NiCl_2_ compared to the controls, the nitrogenase activity of the nodules formed by the wild-type strain and *dme*F mutant were determined. Table 2 indicates that the nitrogenase activities per plant for *M. lupulina* inoculated with *dme*F mutant were reduced by ∼44% or 40% compared to that of *S. meliloti* CCNWSX0020 under 100 mg/kg Ni^2+^ or Co^2+^ stress. However, when standardized by nodule wet weight, the rate of acetylene reduction by nodules infected with the *dme*F mutant was not statistically different from nodules infected with the wild-type strain. When rhizobium successfully infected the nodule cells, it would be dyed blue by toluidine blue. The histological organization of nodules showed that the proportion of infected cells (blue-stained N-fixing cells) within the nodule tissue induced by the *dme*F mutant was not significantly lower than that of the wild-type strain (Fig. 6).

**Table 2:** Nitrogenase activities of nodule infected by *S. meliloti* CCNWSX0020 or *dme*F mutant under Co^2+^ or Ni^2+^ stress.

Bacterial strain	Nodule fresh weight (mg plant^−1^)			Nitrogenase activity (nmol h^−1^ plant^−1^)			Nitrogenase activity (nmol h^−1^ [mg nodule mass]^−1^)		
	0 mg/kg NiCl_2_	50 mg/kg NiCl_2_	100 mg/kg NiCl_2_	0mg/kg NiCl_2_	50 mg/kg NiCl_2_	100 mg/kg NiCl_2_	0 mg/kg NiCl_2_	50 mg/kg NiCl_2_	100 mg/kg NiCl_2_
Wild-type strain *dme*F mutant	5.32 ± 0.5^a^ 4.84 ± 0.8^a^	4.30 ± 0.4^a^ 2.72 ± 0.5^a^	3.99 ± 0.5^a^ 2.28 ± 0.3^b^	54.31 ± 4.1^a^ 48.78 ± 4.7^a^	42.24 ± 3.9^a^ 30.50 ± 3.2^b^	41.88 ± 4.5^a^ 23.64 ± 3^b^	9.96 ± 1.7^a^ 9.91 ± 1.1^a^	9.53 ± 1.5^a^ 11.03 ± 1.4^a^	10.28 ± 1.6^a^ 10.09 ± 1.4^a^
	0 mg/kg CoCl_2_	50 mg/kg CoCl_2_	100 mg/kg CoCl_2_	0 mg/kg CoCl_2_	50 mg/kg CoCl_2_	100 mg/kg CoCl_2_	0 mg/kg CoCl_2_	50 mg/kg CoCl_2_	100 mg/kg CoCl_2_
Wild-type strain *dme*F mutant	5.14 ± 0.5^a^ 4.91 ± 0.8^a^	3.82 ± 0.4^a^ 2.50 ± 0.5^a^	2.60 ± 0.5^a^ 1.88 ± 0.3^b^	52.22 ± 5^a^ 50.08 ± 4.9^a^	38.06 ± 3.6^a^ 28.99 ± 3.2^b^	30.41 ± 3.5^a^ 18.32 ± 3.1^b^	10.11 ± 1.6^a^ 10.18 ± 1.4^a^	9.95 ± 1.3^a^ 11.2 ± 1.4^a^	11.53 ± 1.2^a^ 9.57 ± 1.3^a^

**Notes.**

The values indicate the means ± standard error of triplicate samples.

The letters a/b are significant difference (*p* < 0.05) from plants inoculated with *S. meliloti* CCNWSX0020 or *dme*F mutant under nickel or cobalt stress conditions.

## Discussion

*S. meliloti* CCNWSX0020 is a bacterium that is resistant to multiple heavy metals isolated from root nodules of *M. lupulina* growing in mine tailings in the northwest of China (Fan et al., 2011). The genome of *S. meliloti* CCNWSX0020 was sequenced, and some copper and zinc resistance genes have been analysed in previous studies (Li et al., 2012; Lu et al., 2016; Lu et al., 2017). Here, we characterized the mechanisms of cobalt and nickel resistance and the potential of harnessing these mechanisms for phytostabilization.

In a previous study, Ni^2+^ transporter homologues of NreB in *C. metallidurans* 31A were found in the genomes of *S. meliloti* Rm1021, *S. meliloti* AK83 and *S. meliloti* BL225C through comparative genome analysis (Galardini et al., 2011). Recently, *P*_1b_-5 ATPase, which prevents excessive accumulation of iron and nickel in the cytoplasm, has been discovered in *S. meliloti* (Zielazinski et al., 2013). However, we have not found *nre*B (encoding a Ni^2+^ transporter) in the genome of *S. meliloti* CCNWSX0020 in previous work, and there is no difference in NiCl_2_ resistance between the mutant and the wild type when the *nia* gene (encoding a *P*_1b_-5 ATPase) was knocked out. Analysis of the *S. meliloti* CCNWSX0020 genome led to the identification of a *dmeF*-like gene (SM0020-17742). This open reading frame encodes a protein that has 37% similarity to cobalt- and nickel-resistant proteins (DmeF) of *C. metallidurans*. Upstream of SM0020-17742, an ORF SM0020-17737 encodes an RcnR/CsoR family of metal-responsive transcriptional regulators (DmeR). DmeR could negatively regulate the expression of *dme*RF in the presence of nickel and cobalt in *A. tumefaciens*.

RT-PCR showed that the expression profiles of the *dme*R and *dme*F genes in *S. meliloti* were not only induced by cobalt and nickel but were also induced by copper. However, the expression of *dme*R and *dme*F was significantly up-regulated by nickel or cobalt, whereas no induction of other metal ions was observed (Dokpikul et al., 2016). Previous studies showed that the metal-responsive transcriptional regulator, encoded by *dme*R, could combine with the promoter region of *dme*RF and repress the expression of *dem* RF in the absence of metal. In contrast, nickel and cobalt bind to DmeR and inhibit the interaction of this protein with the *dme*R promoter, and thus, transcriptional repression was relieved in *R. leguminosarum* (Rubiosanz et al., 2013). Similar to *A. tumefaciens* and *R. leguminosarum*, the gene *dme*R (SM0020-17737) was located directly upstream of *dme*F (SM0020-17742) and the non-coding sequences between SM0020-17737 and SM0020-17732 contain a conserved inverted repeat (ATA-GG-GTA-CCCCCC-TAT-GC-TAT) overlapping the −10 sequences, similar to the *A. tumefaciens dme*RF promoter. Meanwhile, DmeR (SM0020-17737) exhibits the residues His3, Cys35, His60 and His64 for Ni^2+^ or Co^2+^ coordination and DmeF (SM0020-17742) contains six predicted transmembrane domains, with two conservative motifs HX3H and HX3D at the beginning of TM2 and TM5, and a histidine-rich stretch having Co^2+^ and Ni^2+^ as substrates (Fig. 1). According to the analysis of the mutants generated by homologous recombination, the *dme*F deletion mutant was most sensitive to cobalt and nickel compared to the wild type, the *dme*F deletion mutant was most sensitive to cobalt and nickel compared to the wild type, but there was no difference in the growth between the *dme*F mutant and wild type under Zn^2+^ or Cu^2+^ stresses, showing the critical role of the DmeF transporter in cobalt and nickel resistance in *S. meliloti* CCNWSX0020. The experimental results of heavy metal resistance are not consistent with the RT-PCR (Figs. 2 and 3). A possible reason is that DmeR belongs to the RcnR/CsoR metal responsive transcriptional regulatory family. RcnR is thought to act as a tetramer and bind to one Ni^2+^ or Co^2+^ per monomer (Iwig & Chivers, 2009). RcnR is structurally similar to CsoR, which is the Cu^+^-responsive repressor of the copper efflux gene *cop*A (encoding a Cu^+^/Ag^+^ efflux P1b-type ATPase) (Ma et al., 2009). We speculated that DmeR from *S. meliloti* could bind Cu^+^ in addition to Ni^2+^ and Co^2+^ with high affinity and up-regulated the expression of *dme*F in vitro. But DmeF can only transfer nickel and cobalt from the cytoplasm to outside the cell.

There was a different cobalt/nickel tolerance ability in the agar plate assay, where the growth of the *dme*F mutant was completely inhibited by 0.4 mM CoCl_2_/0.5 mM NiCl_2_, but in the liquid medium growth test, the growth of the dmeF mutant was significantly inhibited by 0.6 mM CoCl_2_/0.8 mM NiCl_2_ (Figs. 3 and 4). The first reason for this phenomenon was that the liquid medium was cultured for 48 h, while solid medium was only cultured for 24 h. Second, the nutrient assimilation rate is favoured in liquid media and agar could reduce nutrient diffusion throughout the medium (Romberger & Tabor, 1971). Thus, the growth rate of bacteria on solid medium was less than that of liquid medium. So we hypothesized that if the incubation time of solid medium was prolonged, the tolerance to cobalt/nickel in these two assays would be similar.

The effective nodules are directly related to nitrogen fixation efficiency and affect the growth of the legume plant. So the application of rhizobium-legume symbiosis systems for host plant growth promotion and heavy metal absorption in metal-contaminated soils have attracted much attention (Zribi et al., 2013). However, high-concentration heavy metals could inhibit rhizobium growth and associate with the host plant. Metal resistance determinants might protect rhizobia and thereby ensure the ability to build an effective symbiosis relationship under heavy metal stress conditions. The shoot and root biomass of *M. sativa* inoculated with the Zn-tolerant strain *S. meiloti* S532 was higher than plants with the Zn-intolerant strain *S. meiloti* S112. Stan et al. (2011) found that the biomass and nitrogen content of clover inoculated with *R. leguminosarum* biovar *trifolii* were increased compared with untreated plants. Although *S. meliloti* CCNWSX0020 displayed resistance to various heavy metals, the cobalt and nickel resistance mechanisms of this strain were not characterized. Moreover, whether metal-resistant genes affect the symbiotic relationship between rhizobia and plants is not clear under heavy metal stress. Our results showed that excess nickel and cobalt indeed reduced the number of functional nodules, which agreed with other reports that rhizobia-legume symbioses were inhibited by excess metal (Sanchez-Pardo, Fernandez-Pascual & Zornoza, 2012). Although some reports indicated that Ni is used as a structural component of urease and hydrogenase (Brito et al., 1994), Co is mainly used as a component of vitamin B12. Processes in the development of some root nodules specifically require nickel and cobalt. The low supply of Ni^2+^ and Co^2+^ may result in increasing hydrogenase and urease activities in leaves and nitrogenase activities in root nodules (Lavres, Franco & Câmara, 2016). The *dme*F gene deletion aggravated the inhibition of nodulation in the presence of nickel or cobalt (Fig. 5). The *dme*F mutant decreased nitrogenase activity of the Medicago plants under nickel or cobalt stress conditions. However, the rate of acetylene reduction by nodules infected with the *dme*F mutant was similar to that of the wild-type strain when the rate of acetylene reduction was standardized by nodule fresh weight. These results suggest that DmeF could relieve the toxicity of nickel/cobalt to free-living rhizobial cells and help to infect host plants but did not participate in the nitrogen fixation process. This was different from copper-resistant determinants. For example, the *lip*A mutant was not only sensitive to Cu^2+^ but also reduced functional nodule numbers, infected cells, leghaemoglobin expression and N fixation in nodules (Hao et al., 2015). These data suggest that *S. meliloti* selected *dme*RF as a general strategy to maintain nickel and cobalt homeostasis in the cytoplasm.

## Conclusion

In this work, two heavy metal resistance genes (sm0020-17737 and sm0020-17742) of *S. meiloti* CCNWSX0020 were identified with high homology to the *dme*RF operon in *Cupriavidus metallidurans*. The *dme*R and *dme*F genes encoded a transcriptional regulator and cation transporter, respectively. Although the *dme*RF of the CCNWSX0020 strain was induced by Cu^2+^, Ni^2+^ and Co^2+^, the *dme*F mutant exhibited more sensitivity to Co^2+^ or Ni ^2+^than the wild type. Also, there was no difference in the growth between the *dme*F mutant and the wild type under other metal stress conditions. Plant experiment results showed that the nodule number of the host plant inoculated with the *dme*F deletion mutant was significantly decreased in the presence of Co^2+^ or Ni^2+^. However, the nitrogenase activities of nodules infected by the *dme*F deletion mutant were not reduced when standardized by the nodule fresh weight. These results indicated that the *dme*F gene confers Co^2+^ or Ni^2+^ resistance to bacteria and does not participate in the symbiosis with the host plant.

##  Supplemental Information

10.7717/peerj.5202/supp-1 Figure S1 Phylogenetic analysis of DmeF Protein sequences of DmeF or their orthologs in each strain were concatenated and used for drawing the tree.

10.7717/peerj.5202/supp-2 Figure S2 The effects of nickel/cobalt on *dme*F mutant and wild type shoot length A. Stem length of *Medicago lupulina* plants under control, nickel/cobalt (50 mg kg^−1^) and 100 mg kg^−1^) stress conditions. of three replicates. B. Root length of *Medicago lupulina* plants under control, nickel/cobalt (50 mg kg^−1^) and 100 mg kg^−1^) stress conditions. The values indicate the mean±S.E. of three replicates.

## Additional Information and Declarations

### Data Availability

The following information was supplied regarding data availability: The dmeRF sequence is deposited at NCBI with the accession number: AGVV01000035.1.
