# Supplementary figures and images for: Nickel and cobalt resistance properties of Sinorhizobium meliloti isolated from Medicago lupulina growing in gold mine tailing
Source: PeerJ. 2018 Jul 10;6:e5202. doi: 10.7717/peerj.5202 (PMC6044271; doi:10.7717/peerj.5202)

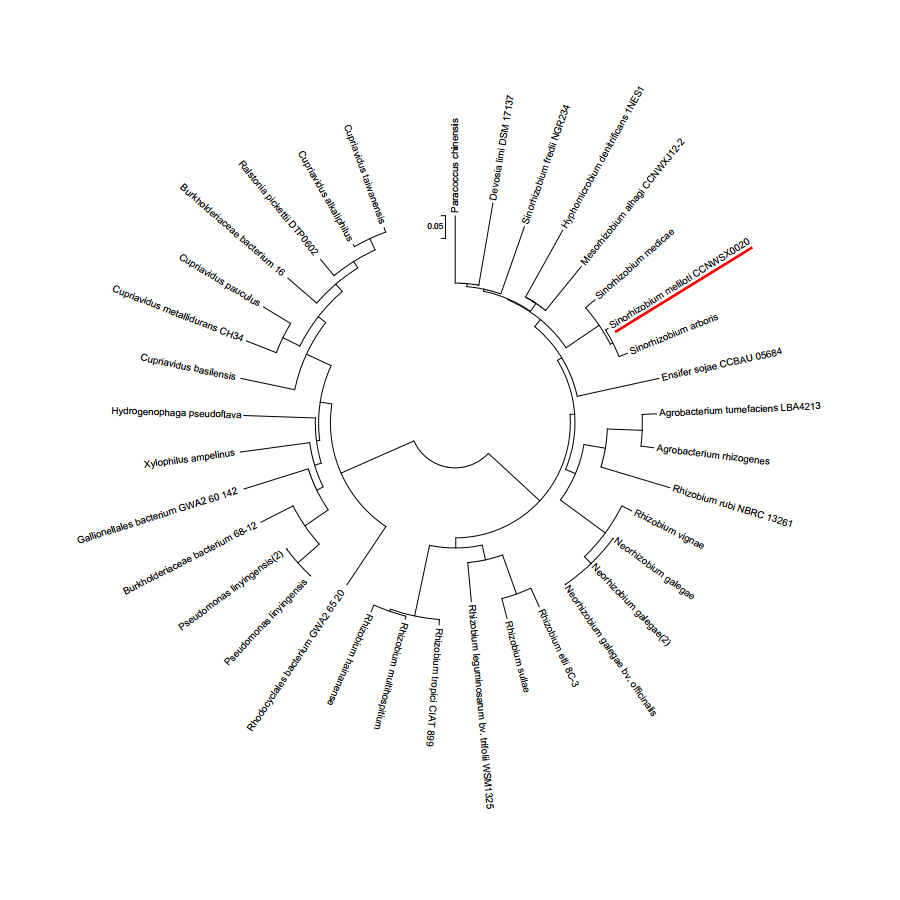

Supplement: Figure S1 — Protein sequences of DmeF or their orthologs in each strain were concatenated and used for drawing the tree. [file peerj-06-5202-s001.png]

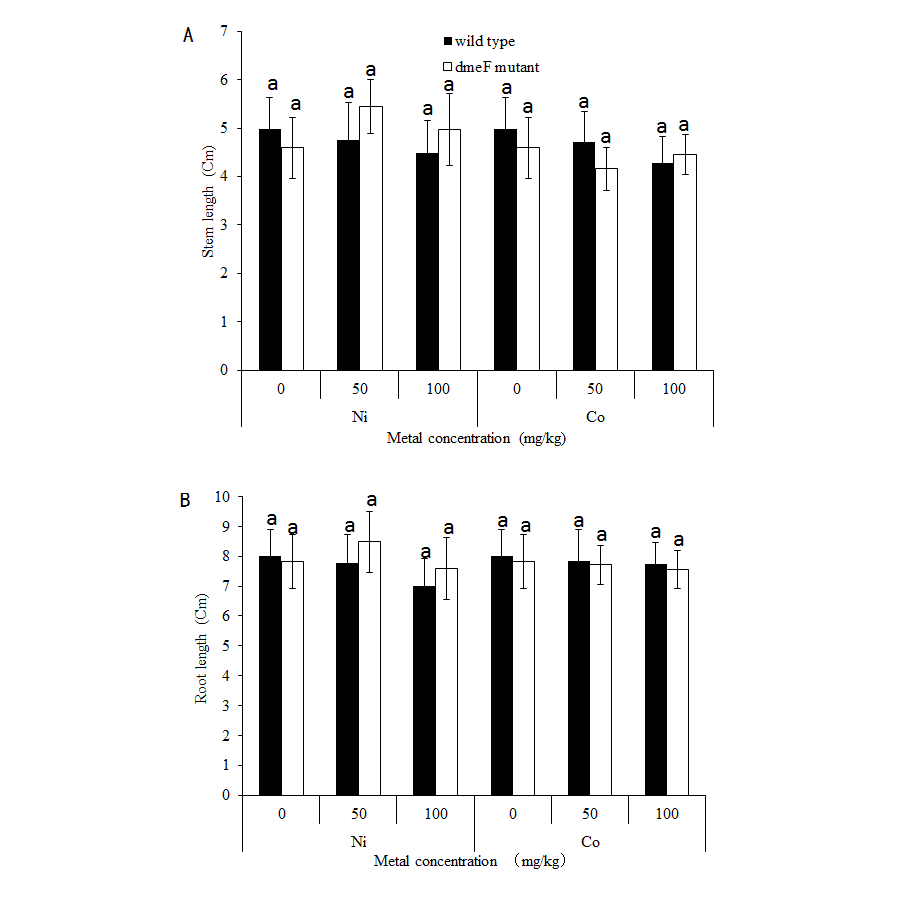

Supplement: Figure S2 — A. Stem length of Medicago lupulina plants under control, nickel/cobalt (50 mg kg−1) and 100 mg kg−1) stress conditions. of three replicates. B. Root length of Medicago lupulina plants under control, nickel/cobalt (50 mg kg−1) and 100 mg kg−1) stress conditions. The values indicate the mean±S.E. of three replicates. [file peerj-06-5202-s002.png]
